# CD2AP at the junction of nephropathy and Alzheimer’s disease

**DOI:** 10.1186/s13024-025-00852-x

**Published:** 2025-06-04

**Authors:** Milene Vandal, Mohsen Janmaleki, Isabel Rea, Colin Gunn, Sotaro Hirai, Jeff Biernaskie, Justin Chun, Grant Gordon, Andrey Shaw, Amir Sanati-Nezhad, Gerald Pfeffer, Frederic Calon, Minh Dang Nguyen

**Affiliations:** 1 Department of Clinical Neurosciences, Hotchkiss Brain Institute, University of Calgary, Calgary, AB T2N 4N1 Canada; 2 Department of Cell Biology and Anatomy, University of Calgary, Calgary, AB T2N 4N1 Canada; 3 Department of Biochemistry and Molecular Biology, University of Calgary, Calgary, AB T2N 4N1 Canada; 4 BioMEMS and Bioinspired Microfluidic Laboratory, Department of Biomedical Engineering, University of Calgary, Calgary, AB T2N 1N4 Canada; 5 Department of Mechanical and Manufacturing Engineering, University of Calgary, Calgary, AB T2N 1N4 Canada; 6 Department of Comparative Biology and Experimental Medicine, Hotchkiss Brain Institute, University of Calgary, Calgary, AB T2N 4N1 Canada; 7 Alberta Children’s Hospital Research Institute, University of Calgary, Calgary, AB T3B 6A8 Canada; 8 Department of Surgery, University of Calgary, Calgary, AB T2N 4N1 Canada; 9 Faculty of Veterinary Medicine, University of Calgary, AB T2N 1N4 Calgary, Canada; 10 Department of Medicine, Division of Nephrology, University of Calgary, Cumming School of Medicine, Calgary, AB T2N 4Z6 Canada; 11 Department of Physiology and Pharmacology, Hotchkiss Brain Institute, Cumming School of Medicine, University of Calgary, Calgary, AB T2N 4N1 Canada; 12 Department of Research Biology, South San Francisco, Genentech, CA 94080 USA; 13 Faculté de Pharmacie, Université Laval, Québec, Québec G1V 0A6 Canada; 14 Centre Hospitalier Universitaire, Laval University, Québec, Québec G1V 4G2 Canada

**Keywords:** CD2AP, Alzheimer, Kidney-brain axis, Brain-body interactions, Neurodegeneration, Sexual dimorphism, Cognition, Cerebrovascular function, Aβ, Tau

## Abstract

Polymorphisms in the gene encoding CD2-associated protein (CD2AP) are associated with an increased risk for developing Alzheimer’s disease (AD). Intriguingly, variants in the gene also cause a pattern of kidney injury termed focal segmental glomerulosclerosis. Recent studies have investigated the cell types and mechanisms by which CD2AP gene dosage contributes to the key pathological features of AD. This review summarizes the fundamental roles of CD2AP in mammalian cells and systems, discusses the novel pathogenic mechanisms focused on CD2AP in AD and highlights the necessity of incorporating biological sex in CD2AP research. Finally, the article draws important parallels between kidney and brain physiology based on vascular and molecular organization, links kidney disease to AD, and suggests the existence of a kidney-brain axis in AD centered on CD2AP.

## Background

Alzheimer’s disease (AD) neuropathology is characterized by intracellular neurofibrillary tangles composed of the microtubule-associated protein tau, and by the extracellular accumulation of Aβ plaques. For decades, most AD studies and therapeutic strategies have focused on these two pathological hallmarks. However, very little is known about CD2-associated protein (*CD2AP*), a top 10 AD genetic predisposition factor [1] with unique biology and therapeutic potential.

CD2AP was initially discovered as a key molecule for the formation of the immunological synapse, thereby promoting the adaptive immune response [2]. Functional genetic studies demonstrated that mutations in the *CD2AP* gene cause a pattern of kidney injury termed focal segmental glomerulosclerosis [3]. How CD2AP contributes to AD is poorly understood. The link between AD and nephropathy caused by *CD2AP* genetic variants also remains a mystery.

In this review, we summarize the fundamental roles of CD2AP in mammalian cells and systems. We then discuss the novel pathogenic mechanisms centered on CD2AP that underlie AD and highlight the importance of incorporating the biological sex factor in CD2AP research. Finally, we draw fundamental parallels between CD2AP-dependent mechanisms in AD and kidney disease, and propose a kidney-brain axis centered upon vascular CD2AP in AD.

### Keypoints

- *CD2AP* genetic variants are associated with both nephropathy and key pathological hallmarks of Alzheimer’s disease (AD)
- Gene dosage studies identify new roles for CD2AP in cognition, neuronal plasticity, parenchymal inflammation and sex-dependent brain vascular function in the context of AD
- The functions mediated by CD2AP in the brain parallel those in the kidney, suggesting shared pathogenic mechanisms and potential therapeutic targets within the kidney-brain axis
- The implication of CD2AP in AD must be analyzed through the perspective of brain-body interactions and taking into consideration of sex differences

## Introduction

In 1998, Dustin and colleagues identified CD2-associated protein (CD2AP) as an important player in the formation of the immunological synapse [2]. This type of specialized junction promotes the interaction between the T cell and the antigen-presenting cell to mount the adaptive immune response. In this process, CD2AP polarizes the cytoskeleton, favors CD2 clustering through direct protein–protein interactions and segregates specific molecules within and from the point of contact between the two cells [2]. Building on this seminal work, CD2AP is now recognized as an adaptor/scaffolding protein that performs a wide range of functions including receptor endocytosis and trafficking to cell–cell interactions, signal transduction, cell adhesion and motility, etc. Importantly, mutations in the human gene are associated with a rare type of kidney injury known as focal segmental glomerulosclerosis (FSGS) [4–6]. Functional genetic studies in mice further demonstrate a critical role for CD2AP in the stabilization of the slit diaphragm, a specialized type of tight junction composed of the interdigitating foot processes of podocytes that acts as a filtration barrier in the kidney [3, 7–9]. While renal CD2AP has been researched extensively, the role of the adaptor molecule in the CNS had remained enigmatic for a decade. The initial interest in brain CD2AP was sparked by the identification of genetic variants in the non-coding and coding regions of *CD2AP* in patients with Alzheimer’s disease (AD) via Genome-Wide Association Studies (GWAS). To date, these variants have not been functionally tested in animal models. In recent years, a series of complementary works have clarified and deepened the role of CD2AP in AD neuropathology, cell signaling, cognitive impairment and brain vascular dysfunction using mice with loss and gain of CD2AP function in specific cell types. In this article, we review and link the pathophysiology of AD to the genetics of CD2AP. We discuss the most recent disease mechanisms mediated by CD2AP and highlight the outstanding questions in the field. Based on the similar barrier, excretion and reabsorption functions that exist between the brain and the kidney, we suggest common mechanisms between Alzheimer’s and kidney diseases. Ultimately, we put forward the concept of a kidney-brain axis centered on CD2AP as a novel framework for the study and treatment of AD.

## Alzheimer’s disease

AD, the most prevalent neurodegenerative disease, is characterized by cognitive decline, with devastating impact on daily life activities [10]. Approximately 30% of people over the age of 85 and 5% between the ages of 65 and 74 suffer from AD [11, 12]. Extracellular accumulation of amyloid-beta (Aβ) plaques and intracellular neurofibrillary tangles are the main pathological hallmarks of the disease. Aβ is generated from the misprocessing of the amyloid precursor protein (APP) and accumulates in the brain decades before the manifestation of cognitive symptoms. Tau aggregates are composed of misfolded hyperphosphorylated microtubule-associated protein tau and are also detectable years before the clinical appearance of the disease [10, 13, 14]. It is now also recognized that a majority of patients diagnosed with AD also present with cerebral vascular pathology which independently contributes to cognitive decline, with its effects adding to those of core proteinopathies (Aβ, tau) [10, 13, 15, 16]. Therapies centered around preserving cholinergic synaptic activity remain the most widely utilized but are largely limited to providing mild symptomatic relief [17]. In an effort to provide disease modification, recent clinical trials show that monoclonal antibodies targeting Aβ are efficient at removing Aβ plaques as assessed with positron emission tomography [18, 19]. While significant clinical benefits were observed, treated patients continued to decline cognitively [20, 21]. So far, few studies target tau pathology, but antisense oligonucleotides directed against tau were shown to inhibit its gene expression on a small cohort of patients with no serious adverse effects [22, 23]. Knowledge gathered from these studies suggest that to truly go beyond symptomatic effects, treatment combinations that target multiple key aspects of AD will likely be necessary [24–26].

Although several modifiable and non-modifiable risk factors have been identified for AD [27]**,** genetic factors play a pivotal role in the development of AD. Twin studies revealed that heritability accounts for ~ 70% of AD cases [28–30]. The genotype for *APOE* is the principal genetic risk factor for sporadic AD [31]. In humans, there are three isoforms for ApoE ((ε2, ε3 and ε4) and each person carries 1 or 2 of these subtypes [31]. People who are homozygous for the ε4 allele have an increased risk of developing AD (up to 15X) while ApoE2 carriers are protected from the disease [32, 33]. Despite the fact that *APOE4* alleles account for 50% of AD cases [34], many other genetic factors influence AD risk. It is also important to highlight that several risk factors associated with a higher incidence of AD simultaneously affect cardiometabolic health and cerebral vasculature [27, 35], emphasizing a high probability of shared pathophysiological mechanisms. Taking into account the vascular localization of CD2AP and its signaling in the brain [36], this review will focus on *CD2AP*, one of the top 10, yet less studied AD-associated genes (www.alzgene.org).

## Genetic of CD2AP in Alzheimer’s disease

An association between *CD2AP* polymorphism rs9349407 and AD was initially found in two GWAS studies [1, 37]. Studies with smaller sample sizes were not able to replicate these findings [38–43]. The association was subsequently confirmed by several GWAS studies in European ancestry, African and Asian populations, as well as by meta-analyses [44–47]. Specifically, several single nucleotide polymorphisms (SNPs) were associated with increased risk for AD (see Table 1). The most commonly linked variant in studies is rs9349407; several other identified polymorphic variants are either intronic or intergenic noncoding variants that are nearly always on the same haplotype (R^2^ = 0.9911 for rs9296559; R^2^ = 0.9719 for rs10948363; and R^2^ = 0.9463 for rs9473117 per LDlink [48] accessed April 21, 2025). Another study in an African population identified the rs7738720 SNP near *CD2AP* as a “suggestive” finding although it did not reach genome-wide significance. This variant is not in linkage with the previously identified SNPs.

**Table 1:** Variants in *CD2AP* associated with AD

Variant ID	Variant consequence	MAF (dbSNP)	Clinical significance (ClinVar)	Study type	References
rs9349407	Non-coding (intronic)	0.230662	Not reported	GWAS	[1, 37, 44, 45, 55]
rs9473117	Non-coding (intergenic)	0.235732	Not reported	GWAS	[47]
rs10948363	Non-coding (intronic)	0.226673	Not reported	GWAS	[46]
rs7738720 *	Non-coding (intergenic)	0.029892	Not reported	GWAS	[56]
rs9296559	Non-coding (intronic)	0.232476	Not reported	Cohort	[52]
rs116754410	Protein-coding (p.Lys633 Arg)	0.0007594	Benign/likely benign	Cohort	[54]

Abbreviations: *GWAS* Genome wide association study, *MAF* Minor allele frequency

^*^ non-significant finding

Subsequent studies attempted to follow up on findings from GWAS. Genotyping of 58 SNPs previously associated with AD, in a study of 547 participants (late onset AD and controls), showed the *CD2AP* rs9349407 variant was significantly associated with AD [49]. This same variant was associated with increased neuritic plaque burden in a clinicopathologic cohort study [50]. In a two-stage genotyping study, the rs9296559 SNP in *CD2AP* was associated with sporadic AD in a cohort of 1001 participants [51]. *CD2AP* gene expression was reduced in peripheral blood lymphocytes of AD patients compared to healthy controls, suggesting that *CD2AP* loss of function in AD is not limited to the central nervous system [52]. Another sequencing study compared variant load between AD cases and controls in genes that had been associated with AD in prior GWAS studies. This study found that a coding variant in *CD2AP*, p.Lys633 Arg (rs116754410), was significantly more common amongst late onset AD cases (*n* = 330) than controls from European ancestry (*n* = 33,370) in ExAC [53]. However, the overall variant burden analysis was not significant for *CD2AP* [54]. Given that several variants in *CD2AP* have been associated with increased risk of AD, it implicates *CD2AP* as a genetic susceptibility and/or as an interaction with environmental or acquired risk factors for AD. Because most SNPs associated with AD risk are usually inherited together (rs9349407, rs9296559, rs9473117 and rs10948363), it implies a possible risk haplotype in *CD2AP* with a potential small effect size. Given the complexity of the *CD2AP* genetic variants in the risk of developing AD, it is paramount to understand the basic biology of CD2AP in the context of the pathogenic mechanisms in AD.

## Basics of CD2AP

**Fig. 1:**
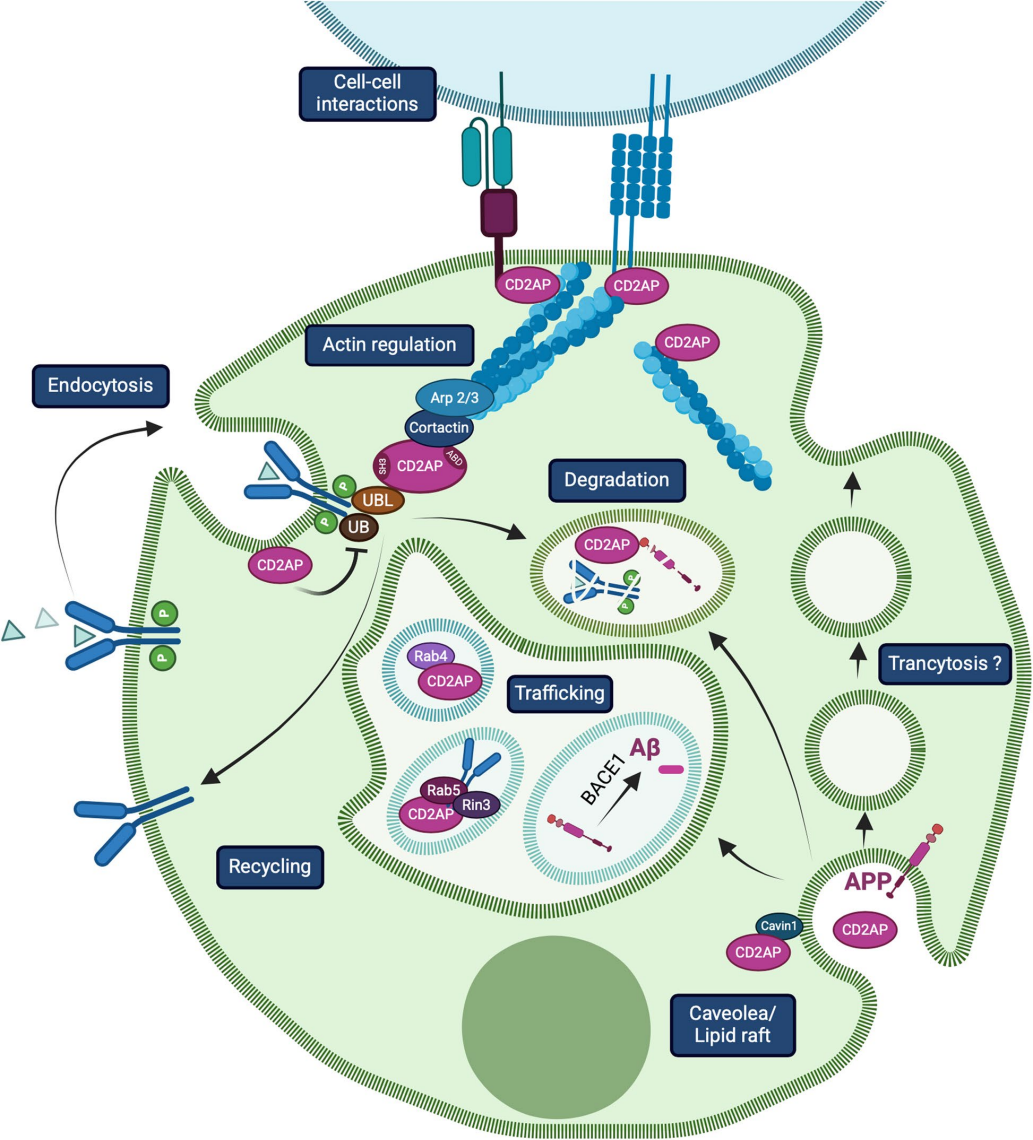
Basic cellular functions of CD2AP. CD2AP plays multiple roles in the cell: 1) the adaptor regulates receptor endocytosis, signaling and degradation via ubiquitin ligases; 2) promotes trafficking of cargoes such as TrkA receptor via Rab GTPases; 3) maintains cell–cell interactions through cell adhesion and tight junction molecules, 4) modulates protein processing and cleavage, as observed for APP, 5) contributes to caveolae formation and 6) triggers cell migration via rearrangement of actin fibers. Some of the CD2AP functions are interdependent such as cell adhesion and cell migration. Most of the functions mediated by the protein involve remodeling of the actin cytoskeleton. Variations and specifics for these mechanisms can be found in the main text

CD2AP is a ~ 70 kDa protein that contains three Src-Homology 3 (SH3) binding domains at the N-terminal, a central proline-rich region, an actin binding site and a coil-coil domain at the C-terminal. The polypeptide is ubiquitously expressed with highest levels in endothelial, epithelial and immune cells [57–62]. Its distribution is often compartmentalized with a preference for the apical or basal side or in specific cellular protrusions such as lamellipodia, pedicels and synapses [58, 63–65]. In buffered-saline solution, recombinant full-length CD2AP forms a roughly spherical structure, consisting of the central coiled-coil domain as a tetramer interface, surrounded by three globular domains corresponding to the three SH3 domains. The tetrameric quaternary structure of CD2AP exposes the binding sites of all 12 SH3 domains, thereby allowing the protein to act as an adaptor, scaffolding and signaling molecule [66]. Its actin binding site at the C-terminal allows for direct interactions with microfilaments [67, 68]. CD2AP also modulates several actin-binding proteins: it interacts with capping protein that limits actin polymerization but also binds cortactin that favors such process [69–73]. Cortactin recruits the Arp2/3 complex to facilitate actin branching while CD2AP can promote Arp2/3 complex-mediated actin assembly through scaffolding of capping protein and cortactin. Binding of CD2AP to capping protein decreases capping activity and also uncaps the actin filament leading to increased actin polymerization and reduced branching. Finally, CD2AP displays inherent capping activity and can therefore effectively shut down fast actin dynamics at filament barbed ends [74]. CD2AP’s ability to remodel the cytoskeleton impacts a wide range of cellular functions and behaviors including receptor-mediated endocytosis/signaling to cargo trafficking, cell–cell interactions, immune response and cell migration (Fig. 1).

### Receptor-mediated endocytosis and degradation

Generally speaking, upon binding of a ligand to its receptor tyrosine kinase (RTK), CD2AP and an associated ubiquitin ligase are recruited to the receptor. CD2AP then signals through its SH3 domains, remodels the microfilament network via actin-binding sites, and then promotes the inward movement of vesicles containing the receptor to be endocytosed. This eventually leads to the ubiquitination and degradation of the receptor to dampen the initial signal or recycling to maintain it (Fig. 1) [75]. RTKs that undergo this CD2AP-dependent regulation (or a variation of it) include the epidermal growth factor (EGF) receptor (EGFR), the vascular endothelial growth factor (VEGF) receptor and the RET receptor in sympathetic neurons [71, 76–78]. Of note, specific receptor activation can lead to the subsequent downregulation of CD2AP indicating the existence of a negative feedback loop [76]. In the context of AD, CD2AP has recently been shown to interact with the RTK colony stimulating factor 1 receptor (CSF1R) and regulates its cell surface levels with implications for hippocampal synapse phagocytosis ([79], see section below). RTKs such as ROR1 that counteracts Aβ toxic effects on the cytoskeleton [80] and microglia TAM receptors that engulf Aβ plaques [81] are candidate targets for CD2AP. Indeed, Aβ processing has been linked to neuronal and microglial CD2AP ([79, 82] see section below). Apart from RTKs, CD2AP also regulates non-RTK receptors such as the innate immunity C-type lectin receptor BDAC2 by preventing its degradation [83], the Intercellular Adhesion Molecule 1 (ICAM-1) adhesion mechanoreceptor [84] by controlling its clustering, and LDL receptor family, such as ApoE receptor 2 (ApoER2, also known as low-density lipoprotein receptor-related protein 8, (LRP8)), in brain endothelial cells to impact brain vascular function ([36]; see section below).

### Vesicular trafficking

CD2AP participates in the intermediate trafficking steps between endocytosis and degradation. The protein associates with specific Rab GTPases and localizes to vesicles marked by these enzymes. Rab GTPases control membrane trafficking, including vesicle formation and movement along actin and tubulin networks, as well as membrane fusion [85, 86]. CD2AP associates with Rab5-bound endosomes and positively regulates TrkA receptor localization to endosomes [63]. Through binding to the Rab5-activating guanine nucleotide exchange factor RIN3 (Ras and Rab interactor 3), CD2AP is also recruited to Rab5-positive early endosomes [87], indicating a role for the adaptor in early endosome trafficking (Fig. 1). CD2AP contributes to the endosomal-lysosomal pathways, notably for APP processing and Aβ generation (see section below). The adaptor also associates with Rab4 in its GTP-bound active form and both localize to vesicular structures in the cytoplasm, along actin fibers and ruffles [88–90]. Both Rab4 and Rab5, are upregulated in neurons microdissected from postmortem brains of individuals with mild cognitive impairment and AD [91] and overactivation of neuronal endosomal Rab5 generates features of AD in mice, including cholinergic neurodegeneration and hippocampal-dependent memory loss [92]. These findings link CD2AP function to vesicular trafficking that is compromised in AD (see sections on AD).

In parallel, CD2AP may also participate in the internalization of membrane-bound molecules through lipid rafts and/or caveolae (a pool of lipid rafts) invagination [93]. Rafts are specialized microdomains in the plasma membrane rich in cholesterol and glycosphingolipids that serve to compartmentalize protein-lipid complexes for signaling and endocytosis [94]. It is also a site where APP and beta-site APP cleaving enzyme 1 (BACE-1) secretase interact to produce Aβ [95]. CD2AP interacts with raft proteins such as podocin in podocytes of the kidney [96] and Cavins to recruit caveolae-associated proteins [93]. These findings suggest that deregulation of CD2AP may contribute to trafficking defects in both AD and nephropathy.

### Cell–cell interactions

At the immunological synapse between the T cell and the antigen-presenting cell, CD2AP has been shown to polarize the actin cytoskeleton, favouring CD2 clustering and segregation of specific molecules within the point of contact between the two cells, thereby promoting the adaptive immune response [2]. CD2AP is also involved in natural killer (NK) cell cytotoxic processes [97]. CD2AP co-localizes with FasL-containing lytic granules in the human NK92 cell line, and is involved in transport of these granules to the NK-target cell contact site for subsequent release/killing [97]. During inflammation, CD2AP regulates migration of plasmacytoid dendritic cells [98]. Levels of CD2AP are decreased in peripheral blood lymphocytes from AD patients, suggesting that its loss of function may compromise the immune response of these cells [52]. Furthermore, the migration of peripheral immune cells to the brain parenchyma through a compromised blood–brain barrier, a phenomenon that is observed in AD, may be linked to CD2AP: CD2AP stabilizes cell–cell adherens junctions [74, 99] and increased blood–brain-barrier permeability is observed in CD2AP knockout mice [100]. The function of CD2AP in peripheral immune cells may be extrapolated to microglia that mediate immune surveillance and synaptic pruning in the brain parenchyma. Similarly, CD2AP in astrocytes and at pre and postsynaptic sites may be important for the regulation of the tripartite synapse [101, 102].

With this general understanding of CD2AP functions in hand, we will now specifically review the novel findings on CD2AP in Aβ pathology, tau toxicity, cognitive and brain vascular functions. We will then present data on the physicomechanical properties of CD2AP-depleted cells and discuss how alterations in these properties are relevant to AD pathogenesis.

## CD2AP in the Aβ hypothesis

Despite the limited impact on disease progression [24–26], targeting Aβ in clinical trials reinforced the Aβ cascade hypothesis for AD [103, 104]. Substantial resources have been and continue to be devoted to study the generation, oligomerization and fibrillation of Aβ toxic species (particularly Aβ42) and clearance of the peptide from the brain [105–108]. In a Neuro-2 A neuronal cell line expressing AD-linked Swedish mutation APP695, suppression of CD2AP by shRNA reduces membrane-bound APP and decreases Aβ40, Aβ42 and Aβ42/Aβ40 ratio in the extracellular media [109]. Compatible with this view, a lower Aβ42/Aβ40 ratio was detected in the brain of 1-month-old female APP/PS1 mice null for CD2AP [109]. It has also been shown that CD2AP together with the synaptic endocytic vesicle protein Bin1 (another AD risk factor [110]) segregate APP from its cleavage enzyme BACE1 in early endosomes of axons and dendrites, thereby preventing Aβ accumulation in neurons [111]. This process is modulated by RIN3 that recruits both CD2AP and Bin1 to early endosomes [112]. Furthermore, overexpression of CD2AP promotes the degradation of APP in lysosomes [113]. On the one hand, CD2AP haploinsufficiency has limited impact on Aβ accumulation in the APP/PS1 mouse model of amyloidosis [109], raising some doubt as to whether endocytic Aβ production from brain cells is the defining role of CD2AP in AD. On the contrary, a recent study showed that promoting the interaction between APP and CD2AP via introduction of a lactyl-mimicking mutation in the APP protein (K612 T) accelerates the endosomal-lysosomal degradation pathway of APP, reduces the load of Aβ produced in neurons and even slows down cognitive deficits in a APP23/PS45 double-transgenic mouse model of amyloidosis [114]. Whether CD2AP is an absolute requirement for the protective effect of K612T remains to be determined. Combined with data showing that APP lactylation is reduced in human AD, these results suggest that promoting APP lactylation may be a promising therapeutic approach for the disease [114]. Consistent with the implication of CD2AP in Aβ biology, a human study using 725 subjects from the Religious Orders Study and Rush Memory and Aging Project shows that *CD2AP* polymorphism is related to neuritic plaque pathology [115]. Further investigation is required to clarify the role of neuronal CD2AP in Aβ toxicity.

CD2AP expressed in other cell types than neurons may also modulate Aβ. A recent study shows that CD2AP is highly expressed in cultured microglia, yet these cells are reactive and stressed when isolated and cultured. CD2AP levels are augmented in AD brains as per proteomic analysis of a published database [79]. Furthermore, CD2AP haploinsufficiency slows down the transition from homeostatic to disease-associated microglia state in response to Aβ in the 5xFAD transgenic mice. Ultimately, CD2AP-deficient microglia attenuate hippocampal synaptic loss in the AD mouse model presumably by reducing the expression of C1q complement that marks synapses for phagocytosis via Colony stimulating factor 1 receptor [79]. Intriguingly, multiple single-cell RNA seq datasets show very low levels of *CD2AP* in mouse microglia compared to other mouse brain cell types (such as neurons and brain endothelial cells, [116, 117]). Consistently, several studies including ours did not detect significant CD2AP expression in mouse microglia using immunofluorescent staining and confocal microscopy [36, 58, 60, 82, 118]. In humans, *CD2AP* mRNA is highly expressed in microglia ([102, 119], https://brainrnaseq.org). Of note, Zhang and colleagues used a proteomic human database to detect protein upregulation in human AD brains but that is not restricted to microglial *CD2AP *expression. Further, they did not confirm in situ expression of *CD2AP* in microglia for either their WT or AD mice. It is therefore unclear how differences in the expression and function of human vs. mouse microglial *CD2AP* may influence its role in AD. It also remains unclear why haploinsufficient and not homozygous microglia knockout mice were used for the study. Homozygous microglia knockout mice generated in the Shaw lab are viable and show no overt phenotype (personal communication). As microglia are highly dynamic cells with populations varying over disease course, microglial CD2AP may play protective and/or detrimental roles depending on the stage of the disease. Combined with a characterization of *CD2*AP expression in microglia of WT and AD mice at different ages, the generation of conditional microglia-specific *CD2AP* KO would be an asset to answer these outstanding questions.

**Fig. 2:**
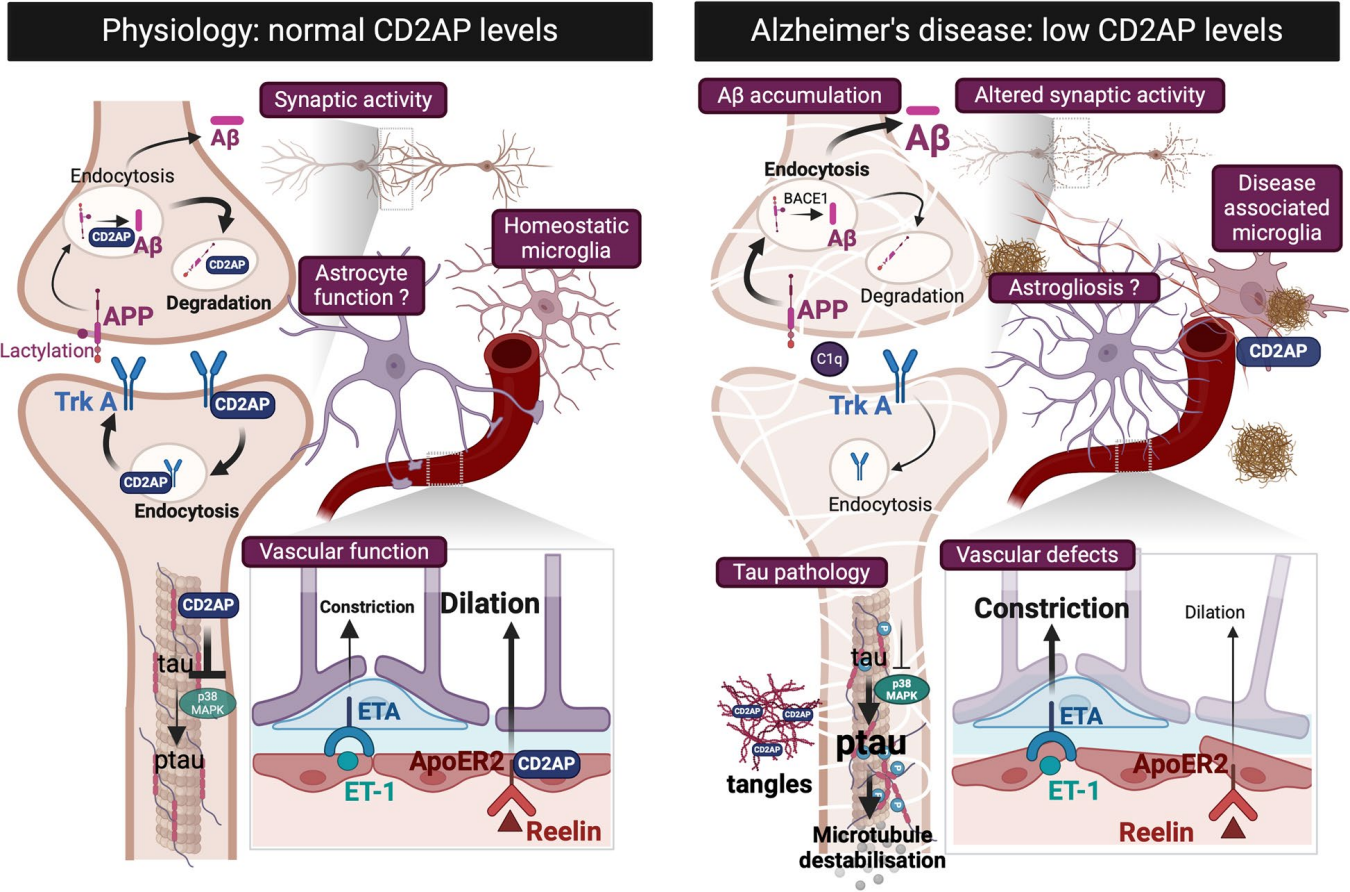
Cell type-specific roles of CD2AP in the pathogenesis of Alzheimer’s disease. Loss of CD2AP function in specific cell types produce features of AD at the molecular, cellular, behavioral and organismal levels. Neuronal CD2AP segregates APP from BACE1, thereby preventing the accumulation of Aβ; it also promotes APP degradation in lysosomes. Enhancing the interaction between APP and CD2AP by lactylation of the former favors the endosomal-lysosomal degradation pathway of APP and overall, reduces the burden of Aβ produced in neurons (left panel). Loss of neuronal CD2AP affects these processes, thereby resulting in the accumulation of Aβ in mouse model of amyloidosis, with implication for fibrils and plaques formation (right panel). Absence of neuronal CD2AP also promotes the hyperphosphorylation of tau by p38 kinase causing microtubule destabilization and synaptic defects. CD2AP forms neuronal inclusions similar to neurofibrillary tangles and neuropil thread‐like deposits in post-mortem AD samples where it co-localizes with hyperphosphorylated tau (right panel). The trafficking and recycling of the neurotrophic/neuroprotective TrkA receptor may be also impaired upon loss of CD2AP, contributing to loss of cholinergic neurons. Tau propagation may also be impacted by the genetic variants of *CD2AP* in neurons. Conversely, microglial CD2AP appears to favor the progression of homeostatic to disease-associated microglia (DAM) (right panel). Low levels of microglial CD2AP appear to restrain these cells in their homeostatic state by preventing the marking of synapses with C1q complement (to be phagocytosed) and attenuates synaptic defects in the 5XFAD mouse model. Whether mouse and human microglia mediate similar function with populations varying over disease course remain to be determined. Finally, endothelial CD2AP regulates brain vascular function in a sex-dependent manner by balancing the vasoconstriction and vasodilation through ET1/ETA ligand/receptor complex and Reelin/ApoER2 signaling, respectively. In AD, this balance is perturbed with a predominance for the vasoconstrictive effects and such alteration is tightened to loss of brain endothelial CD2AP. Loss of brain endothelial CD2AP triggers memory deficits in mice

*CD2AP* is highly expressed in the brain endothelium of both mice and humans [36, 58, 68, 116, 120–123]. Interestingly, loss of CD2AP in brain endothelial cells does not impact amyloidosis in the parenchyma nor does it trigger the accumulation of Aβ around vessels (a diseased condition called cerebral amyloid angiopathy that is observed in more than 90% of AD cases) in the PS2 APP mouse model of amyloidosis [36]. However, the reactivity of vessels to Aβ is affected in the absence of CD2AP in these cells, as evidenced by two-photon microscopy studies in awake mice (see section on the brain vascular function and [36]). In sum, there is evidence for a role for CD2AP in Aβ toxicity and pathology, but which cell types are reliant on CD2AP and how exactly CD2AP regulates Aβ biology in specific models of amyloidosis and AD remain to be fully investigated (see Fig. 2).

## CD2AP entangled with tau

Tau post-translational modifications, mRNA splicing alterations and cellular stress can disturb the microtubule-associated function of tau, leading to its aggregation and propagation in AD [124]. A few studies have linked CD2AP to tau toxicity. In Drosophila, genetic deletion of *cindr*, the fly ortholog of *CD2AP*, exacerbates human tau-mediated neurodegeneration [125]. This phenotype may be related to the functions of Cindr in synaptic transmission, calcium homeostasis and protein degradation (see section below on synaptic activity). In the APP/PS1 mouse model of amyloidosis, Xue and colleagues reported that neuronal knockout of CD2AP exacerbates tau hyperphosphorylation, synaptic impairments and cognitive deficits [82]. The increased tau pathology in these mutant compound mice involves the activation of p38 mitogen-activated protein kinase that is known to hyperphosphorylate tau (Fig. 2). Pharmacological inhibition of the kinase reduces neuronal apoptosis and even improves the synaptic and cognitive defects [79]. As tau toxicity correlates with synapse loss and neuronal cell death, these results suggest that CD2AP is an effector in neuronal demise in AD. Compatible with this finding, p38-JNK is emerging as a viable target for AD based on its identification in several experimental systems [126, 127]. Thus, whether defects in CD2AP lead to the accumulation of tau and contribute to tauopathies warrants future studies.

There is also genetic evidence linking CD2AP with tau. Interestingly, in a cohort of 672 European participants, CSF total tau and not Aβ is associated with *CD2AP* polymorphism rs9349407 [128]. Furthermore, two additional studies including patients from the Religious Order Study (ROS), the Rush Memory and Aging Project and the Alzheimer’s Disease Neuroimaging Initiative confirm that *CD2AP* SNPs rs9381563 and rs10948363 are linked to higher CSF ptau and neurofibrillary tangles [129, 130]. CD2AP also forms neuronal inclusions in a manner reminiscent to neurofibrillary tangles and neuropil thread‐like deposits in post-mortem AD samples where it co-localizes with hyperphosphorylated tau [118]. In these samples, no co-localization was found between neuronal CD2AP and Aβ parenchymal plaques as well as between vascular Aβ and vascular CD2AP. Moreover, neuronal CD2AP accumulates in a hierarchical progressive fashion from the entorhinal to the temporal and occipital cortex, typical of AD. Its neuronal immunoreactivity was strongly associated with Braak stage and this was observed independently of age and other AD pathological hallmarks [118]. The association of CD2AP neuronal expression was detected in 3 repeat‐tau‐diseases such as AD and Pick’s disease but not 4-repeat-tauopathies including progressive supranuclear palsy and corticobasal syndrome. These findings raise the central question as to whether CD2AP is important for tau propagation, particularly in the late stage of disease where the 3-repeat isoform predominates [131]. As neuronal activity influences tau spreading [132], the selective correlation between neuronal CD2AP and 3-repeats tau diseases may be related to a defined neuronal activity modulated by CD2AP. We also recently found that in vascular-enriched cortical samples from the Religious Order Study cohort, the levels of soluble CD2P negatively correlated with tangles and phospho-tau whereas the levels of insoluble CD2AP positively correlated with phospho-tau [36]. Overall, these indicate that the association between CD2AP and tau toxicity and pathology might involve multiple mechanisms and cell types. Further studies with human samples are required to fully understand the links between CD2AP and tau in AD.

## CD2AP in synaptic activity

In podocytes, CD2AP functionally interacts with endophilin, dynamin and synaptojanin [133]. These proteins are also active at neuronal synapses, suggesting that CD2AP acts at these cellular sites as it does in podocytes. Notably, murine CD2AP and its ortholog Cindr in Drosophila are active at the post and pre-synaptic terminals, respectively [101, 102, 134]. At the pre-synapse, absence of Cindr impairs synaptic maturation and synaptic vesicle recycling and release, thereby providing the first clue that CD2AP may be important for neurotransmission. In this context, Cindr controls the levels of calcium channels and synapsin. The adaptor also complexes with another adaptor, 14–3-3ζ, to control protein degradation [134], most likely that of synaptic proteins through the ubiquitin–proteasome system. In the mouse brain, CD2AP co-localizes with the pre-synaptic marker synapsin. Paired-pulse facilitation at hippocampal Schaffer-collateral synapses was augmented in mice with one or two germline CD2AP null allele(s) [102], consistent with CD2AP pre-synaptic expression and the haploinsufficient requirement for pre-synaptic release. In addition, CD2AP positively regulates the trafficking of the pro-survival and neurotrophic receptor TrkA [63] that is expressed in cholinergic neurons, the most vulnerable population of neurons in AD [17]. Activation of TrkA by nerve growth factor has been shown to increase the release of acetylcholine under depolarizing conditions [135] and to promote long-term potentiation, a form of synaptic plasticity and proxy of learning and memory ([136], see [137] for a review). At the post-synaptic side, CD2AP tunes the trafficking and processing of APP to control the production of Aβ (see section above, Fig. 2). Processed APP and Aβ have roles in synaptic plasticity [138–143], yet the molecular mechanisms still need to be unraveled. In dendritic spines of cultured neurons, CD2AP remodels the F-actin network, thereby modulating synapse and neuronal activity [101, 102]. Importantly, Nestin-cre driven conditional knockout for *CD2AP* that abrogates gene expression in neuronal progenitors and neurons, and neuron-specific CD2AP knockout mice show no signs of cognitive impairment, even at 12 and 15 months of age, respectively [82, 102]. This absence of phenotype could be due to an adaptation of the neuronal network and/or molecular compensation, as suggested by changes in protein folding, lipid metabolism, proteostasis, synaptic homeostasis and plasticity detected by proteomics in both reports [82, 102]. Alternatively, these studies may suggest that neuronal CD2AP is not the main driver for cognitive function, despite its relevance to synaptic plasticity and neurotransmission.

## CD2AP in cognition

A study of 73 non-demented and demented patients revealed that *CD2AP* polymorphism rs9349407 was associated with a 6% lower Mini-Mental State Examination score, a questionnaire used to measure cognitive impairment and screen for dementia [144]. Verhaaren et al. also found that the same SNP was tightly linked to memory function in a cohort of 5171 non-demented subjects [145]. The relevance of CD2AP to memory dysfunction in AD is further substantiated by human data showing that the post-mortem levels of vascular CD2AP (but not total CD2AP) are lower in AD volunteers, particularly in male individuals, and such decrease is associated with poor cognitive performance, more specifically for the episodic and semantic memory [36]. To understand the significance of this association, two mouse lines lacking *CD2AP* in brain endothelial cells were generated. Contrary to the two neuronal *CD2AP* knockout mouse models [82, 102], the mutant endothelial *CD2AP* mouse lines show memory deficits as assessed by behavioral assays (novel object recognition and the Barnes maze tests, [36]). Consistent with this finding, using functional ultrasound to measure brain connectivity (a key correlate to memory function [146]), perturbations in the connectivity between brain areas involved in navigation were found in these mutant *CD2AP* mice [36]. These mice also exhibit reduced vessel density in the hippocampus and higher markers of microglia and macrophage activation at 15 months of age [36] suggesting that long-term loss of CD2AP might damage brain vessels, increase brain inflammation and contribute to memory impairment. In brief, the impact of *CD2AP* polymorphisms on cognitive function [144, 145] can be, in part, explained by the endothelial protein rather than its neuronal form. In the next section, we will review the role of CD2AP in brain vascular function and explain how this could affect memory function.

### Brain endothelial CD2AP: a new player in AD

Vascular contributions to neurodegenerative diseases—especially AD—are gaining growing recognition. Major AD risk factors such as diabetes, hypertension and carriage of *APOE4* allele, are associated with vascular pathology [32, 147–149]. An estimated ~ 75% of AD patients have vascular abnormalities that include cerebral amyloid angiopathy (CAA), microinfarcts, ischemic lesions and blood–brain barrier dysfunction [150, 151]. Some of these defects are observed in individuals suffering from mild cognitive impairments, a condition that often precedes AD [152]. Effective management of AD, both in prevention and in disease-modifying treatment, may depend on slowing the underlying vascular pathology.

As part of the vascular defects in AD, patients exhibit anomalies in brain endothelial cell markers and transporters, CAA, lower expression of genes linked to angiogenesis as well as altered cerebral blood flow (CBF) regulation [123, 153–163], all in line with the idea of endothelial cell dysfunction. Higher levels of CD2AP in the plasma are also associated with higher risk for AD, perhaps due to leakage from the brain endothelium [164]. Consistent with the critical function of CD2AP in brain vessels, compound mutant *CD2AP* null mice overexpressing the protein in the kidney to bypass early lethality, display a disrupted blood–brain barrier [100]. In humans, polymorphism rs9296551 is associated to fibromuscular dysplasia, a disease characterized by the accumulation of fibrous tissue and webs in arteries that cause narrowing of the vessels [165]. Furthermore, rs9349407 [166] and rs9381563 [130] polymorphisms are associated with reduced brain glucose uptake. The *CD2AP* gene is also linked to left angular gyrus CBF in the Alzheimer's Disease Neuroimaging Initiative cohort [167]. Finally, patients with cerebral small vessels disease have lower expression of the protein [168]. Altogether, these results suggest that alterations in CD2AP are detrimental for brain vessels in AD.

In the mouse and human brain, *CD2AP* expression is predominantly found in brain endothelial cells [36, 58, 68, 116, 120–123]. Acute or chronic loss of brain endothelial CD2AP alters brain microvessel response to whisker stimulation, Aβ application as well as treatment to Reelin, a glycoprotein that dilates vessels and signals through an ApoER2-CD2AP complex [36]. Altered CBF at rest and during neurovascular coupling, the process that links the energetic demand of brain neurons to the supply of nutrients from the blood, are also observed in mice lacking brain endothelial CD2AP in a sex-dependent manner, with more profound effects in male mice [36]. This is caused by the disrupted interface between brain endothelial and mural cells activity assessed by calcium signaling, downstream of the vasoconstrictive action of endothelin-1 (ET1) on ETA receptor [36]. Furthermore, blocking the vasoconstrictive action of ETA, partially rescues vascular impairments in male mutant *CD2AP* mice (Fig. 2). Conversely, promoting the counteracting ApoE receptor 2 (ApoER2)/endothelial Nitric Oxide Synthase (eNOS) pathway with Reelin, yielded benefits in both male and female mutant mice via partial restoration of CBF. The very large decrease (60%) in baseline red blood cell velocity (a proxy for CBF) and the 50% increase in capillary stalls found in the mutant CD2AP mice likely cause cognitive impairment in these mice [36] based on studies reporting memory deficits in models with similar CBF abnormalities [169–172]. Finally, beyond the capillary blood cell data that link cerebrovascular function to cognition, all the other morphological, functional and pathological changes resulting from the loss of CD2AP in brain endothelial cells are likely contributing to their memory impairment. This includes the altered response of vessels to stimuli (whisker stimulation, Aβ treatment), the altered mural cell activity, the brain parenchymal inflammation and decreased vessel density with age, as well as the increased elasticity of CD2AP-depleted brain endothelial cells that can be extrapolated in vivo in the context of shear stress (see section below).

### Sex matters for CD2AP

The findings of sex differences in brain vascular function and cognition related to CD2AP are intriguing.

At the present time, it is still unclear whether *CD2*AP mRNA and protein levels per se are different between males and females. Indeed, transcriptomic databases from human tissue and mouse tissues indicate contradicting findings: Higher levels of CD2AP in females vs males [173] or no difference between sexes [174]. In our recent human study, no difference in soluble CD2AP between male and female subjects was found. Higher content of insoluble vascular CD2AP, specifically in male AD volunteers, was observed. However, there was an association between biological sex and the diagnosis suggesting that the aggregation of the protein might be modulated by these two parameters [36]. In brief, CD2AP contributes to the sexual dimorphism of the brain vascular system at multiple levels, particularly at the interface between brain endothelial cells and mural cells, with impact on AD pathogenesis but the levels of vascular CD2AP per se do not differ by sex. Of note, memory function of female endothelial *CD2AP* mutant mice was not assessed [36]. As human studies found the strongest correlation between low levels of vascular CD2AP and cognitive impairment in male AD individuals [36], male mice were selected for all behavioral analyses. As AD affects a higher proportion of females [175], the role of brain endothelial CD2AP in female subjects must be addressed in the future. It would be equally critical to determine whether CD2AP mediates sex-dependent functions in other cell types such as glia, mural cells and neurons. For example, exposure to the Aβ peptide triggers a heterogenous microglia response characterized by a signature enriched with AD genes, and this response is more rapid in female mice than male mice [176], all consistent with the higher incidence of female individuals in AD [175]. Considering the emerging role of microglial CD2AP in AD, it is important to determine whether CD2AP modulates this differential response between sexes in a temporal manner. In sum, all research on CD2AP must take the factor of biological sex into consideration.

### Physico-mechanical properties of CD2AP-depleted cells

All the studies performed on CD2-AP have focused primarily on the cellular and molecular aspects of cells, tissues and organs. However, assessing the physico-mechanical properties of cells lacking CD2AP is vital to understand the physiological role of CD2AP. These properties are likely to be altered in AD where cytoskeletal disruption, loss and/or abnormal cell–cell interactions (tripartite synapse, microglia-neuron interactions, neurovascular unit interactions), and deleterious cell-molecules interactions (Aβ with neurons, microglia, endothelial cells) are found [177].

**Fig. 3:**
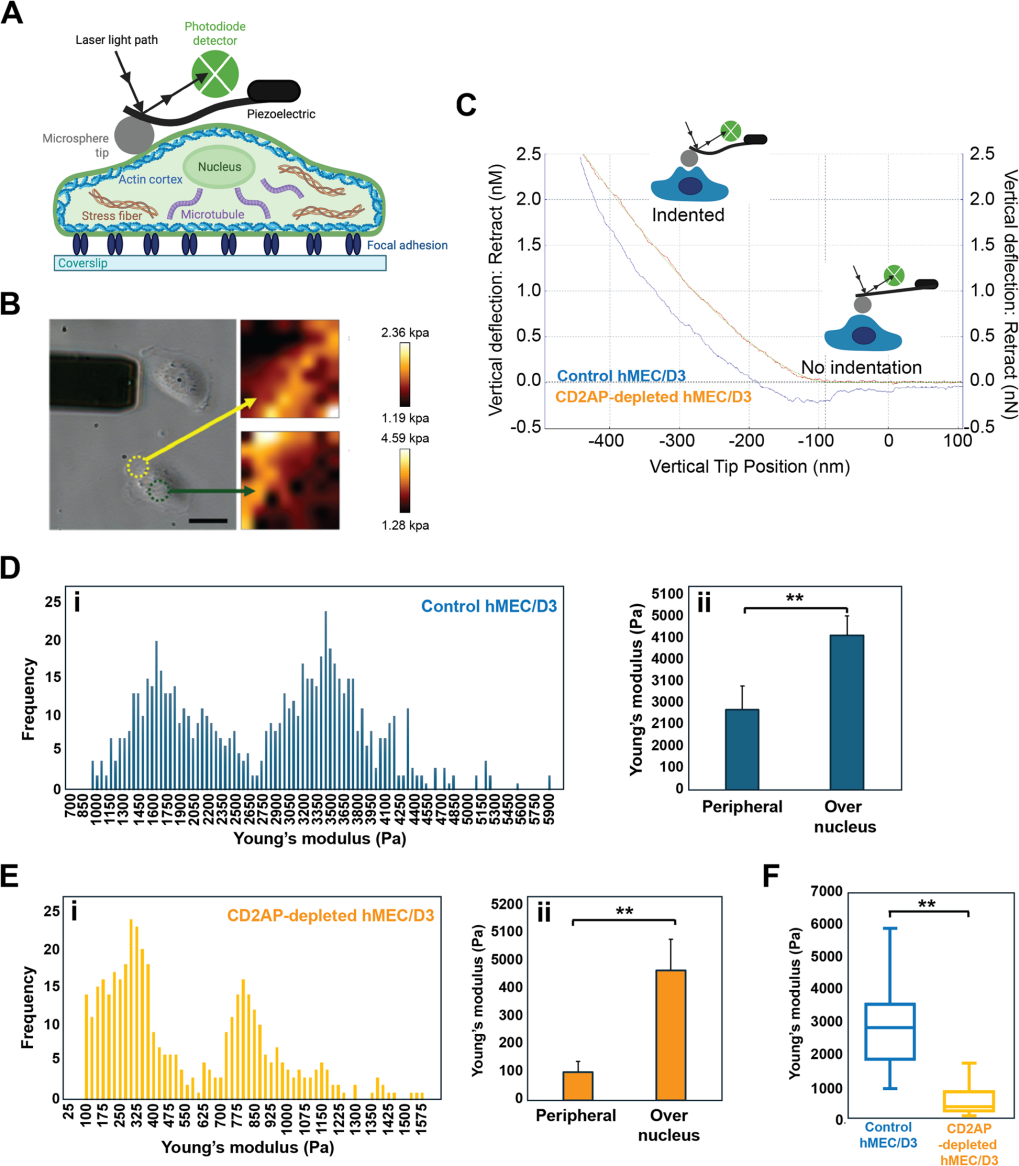
Physico-mechanical properties of immortalized human brain endothelial cells (hCMEC/D3) depleted of CD2-AP determined by atomic force microscopy (**A**) Schematic of the AFM setup used to measure cell stiffness via shallow indentation with a microsphere-tipped cantilever. The system detects deflection to estimate mechanical properties of subcellular regions, including the actin cortex, nucleus, and focal adhesions. **B** Optical image of an hCMEC/D3 cell under the AFM cantilever (left), with corresponding stiffness maps (right) showing local Young’s modulus values in the peripheral region (green arrow) and over the nucleus (yellow arrow). Stiffness values ranged from 1.19–2.36 kPa peripherally and 1.28–4.59 kPa above the nucleus. **C** Representative force-indentation curve with the red line indicating the fitted Hertz/Sneddon model used to calculate Young’s modulus from the approach curve; the green line shows the raw indentation curve, and the blue line corresponds to the retraction curve. Maximum indentation was limited to 500 nm to minimize cytoplasmic contributions. **D** Distribution of Young’s modulus values in control hCMEC/D3 cells: (i) Histogram showing frequency distribution across all sampled points; (ii) Bar graph comparing stiffness between peripheral regions and regions above the nucleus (*p* < 0.05, two-tailed unpaired t-test). **E** Distribution of Young’s modulus in CD2AP-depleted hCMEC/D3 cells: (i) Histogram shows a leftward shift in stiffness values compared to controls; (ii) Bar graph demonstrates significant stiffness reduction both peripherally and over the nucleus (*p* < 0.05, two-tailed unpaired t-test). **F** Box-and-whisker plot comparing overall cortical stiffness in control versus CD2AP-depleted cells. *CD2AP* knockdown significantly reduced Young’s modulus across both regions (*p* < 0.05, two-tailed unpaired t-test).

Using atomic force microscopy, spherical tips attached to the cantilevers to induce nanoindentations in the cell membrane and calculations with the Young’s modulus (an index of cell elasticity/stiffness), we found that brain endothelial cells depleted of CD2AP display reduced stiffness/increased elasticity (Fig. 3). In the context of brain vascular function and blood–brain barrier permeability, these results suggest that CD2AP-depleted cells would respond differently to shear stress from changes in blood flow as well as to the toxic accumulation of proteins such as Aβ and tau in vivo. As CD2AP plays a fundamental role in organizing the actin cytoskeleton and actin depolymerization by shear stress increases endothelin 1 (ET-1) [178], the enhanced elasticity of CD2AP-depleted brain endothelial cells may contribute to the imbalance in ET-1 vs Reelin-eNOS signaling observed in the mutant endothelial *CD2AP* mice, and consequently in the vasoconstriction/vasodilatory imbalance [36].

The changes in cellular flexibility of brain endothelial cells depleted of CD2AP may synergize with the toxicity of Aβ and tau to destabilize the actin cytoskeleton [143, 179]. Actin dynamics and anisotropic tension play a central role in governing endothelial mechanics. Increasing luminal pressure with a microstretcher system induces a fluid-like expansion and directional reorganization of actin fibers, thereby causing endothelial tissues to transition from isotropic to anisotropic tension states [180]. A new mechanical model, treating actin as a nematic network coupled with active stresses provides a novel framework to understand how cytoskeletal organization directly modulates vascular mechanical properties under physiological and pathological conditions.

The alteration in the rigidity of brain endothelial cells lacking CD2AP can be further extrapolated to other cell types that express CD2AP. For example, a change in the flexibility of microglia expressing *CD2AP* polymorphism could compromise their immune response, phagocytic, pruning activities and clearance of Aβ in the brain parenchyma. Similarly, the dynamics of tripartite synapse and neurovascular unit may be disturbed by these SNPs, leading ultimately to altered neurotransmission/excitotoxicity and cerebral vascular/blood–brain barrier dysfunction, respectively. In sum, understanding the physico-mechanical properties of cells will shed new light in our interpretation of the multiple roles of CD2-AP in AD. Such consideration may also explain why CD2-AP genetic variants affect both the kidney and brain, two organs continuously subjected to high blood flow.

For B-F, elasticity is calculated from force-indentation curves obtained from AFM probe exertion onto the cells’ cortex (*N* = 1130 curves, *n* = 28 cells). The curves were fitted to the model, in which the tip and the cell are assumed isotropic and homogeneous with axisymmetric and continuous contact geometry. The equation for spherical tip which relates force and depth for indentation is given by:

$$F=\frac{4E}{3(1-{v}^{2})}\sqrt{R{\delta }^{3}}$$

where *ν, F, δ, E, and R* are the Poisson’s ratio, loading force, indentation, Young's modulus, and radius of the curvature of the AFM tip respectively.

### CD2AP in the kidney-brain axis?

Emerging studies suggest a strong connection between nephropathy and neurodegeneration via the circulatory and immune systems and renal afferents. If left untreated, nephropathy can progress to chronic kidney disease (CKD) that includes FSGS and, eventually causes kidney failure (also known as end-stage renal disease). CKD patients develop cognitive dysfunction and dementia while memory impairment and dementia, in turn, can increase the mortality of patients with CKD [181–186]. To date, there is no clear indication that CD2AP genetic variants in FSGS cause AD. In described cases of renal disease due to *CD2AP* variants, the age of onset is generally quite young (ages 2–21 [187]), and it is possible that with prolonged follow-up these individuals may eventually develop pathology consistent with AD. It is interesting to note the AD-related coding variant in *CD2AP* p.Lys633 Arg (rs116754410) corresponds to a highly conserved amino acid position in the coiled-coil domain of the protein near the C-terminus. When comparing this variant to those described with glomerulosclerosis, one of them is a stop-gain variant at proximity of the coiled-coil domain. This comparison suggests that some cases of nephropathy and AD may converge mechanistically through CD2AP. Furthermore, ApoE, clusterin and Sortilin Related Receptor 1 (SORL1) that are implicated in APP processing and Aβ toxicity and clearance in AD, have been proposed as candidate genes for the memory deficits in patients with CKD [188].

**Fig. 4:**
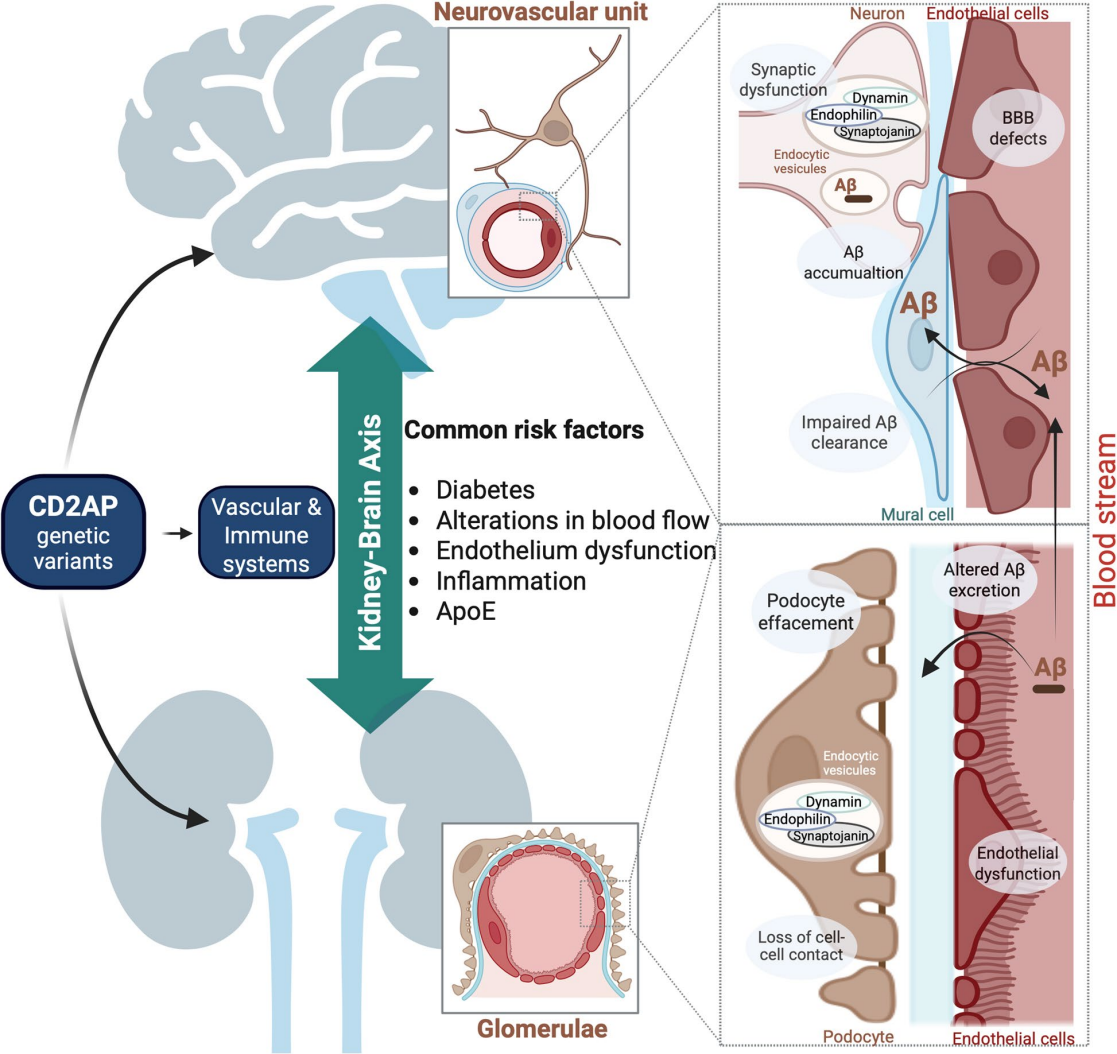
A putative kidney-brain axis centered on CD2AP. Similarities between the kidney and the brain at the level of cellular, structural and functional organization: 1) slit diaphragm vs blood–brain barrier; 2) podocytes vs neurons with overlapping molecular composition (dynamin, synaptojanin, endophilin and 3) glomerular endothelium vs brain endothelium. In both organs, CD2AP plays key roles in a cell-type specific manner. Genetic variants in CD2AP gene cause kidney injury termed focal segmental glomerulosclerosis or AD, and patients with CKD develop memory impairment. Podocytes effacement can be related to loss of and/or dysfunctional synapses. The enrichment of CD2AP in cerebral and glomerular endothelia may be the reason why both organs are vulnerable to *CD2AP* genetic variants: if weakened, endothelia of both organs may be further damaged by high-volume of blood flow through the cardiac cycle. Other risk factors include diabetic conditions and ApoE genotype that has been linked to *CD2AP* polymorphism, brain vascular and renal functions. While brain CD2AP is important for Aβ production and potentially clearance, the kidney has the ability to clear circulating Aβ but the organ may fail in doing so in disease conditions. Indeed CKD patients display elevated levels of serum Aβ, enhanced deposition of Aβ in the brain and lower Aβ CSF levels. Taken together, a bidirectional kidney <—> brain axis centered on CD2AP may exist and be altered in AD

Structurally and functionally, there are similarities between the kidney and the brain (slit diaphragm vs blood–brain barrier; podocytes vs neurons; glomerular endothelium vs brain endothelium, see Fig. 4). The enrichment of CD2AP in endothelia might explain why both organs are affected by *CD2AP* genetic variants. Indeed, both organs act as filtration and protective barriers, are densely vascularized and consequently, vulnerable to small vessel dysfunction [189]. The blood–brain barrier also shares homologies with the Bowman’s Capsule, glomerular basement membrane and slit diaphragm at the organizational and functional levels, and both are altered upon CD2-AP loss. Indeed, CD2AP is expressed in both the fenestrated glomerular and tight cerebral endothelium, serving a similar barrier function for reabsorption (influx) and excretion (efflux). Compound mutant CD2AP mice display blood–brain barrier leakage [100] while CD2AP straight knockout mice display damaged glomerular endothelium [190]. In addition, mice with depletion of endothelial CD2AP display impaired brain vascular and memory function and with age, reduced vessel density [36].

A possible unifying model is that perturbations of the vascular system caused by CD2AP alterations are a common denominator underlying renal diseases and AD. This notion of a shared risk factor model is particularly attractive when considering that both kidney and brain are low-resistance end-organs, continuously subjected to high volume of blood flow via the cardiac cycle [191]. For instance, patients with CKD have brain vessel microbleeds that are observed in AD [192–197] and present higher risk for stroke, a condition that predisposes to AD [198–202]. Another possible common risk factor between AD and CKD lies in the diabetic condition. High blood glucose is responsible for one third of CKD in the adult via damaged blood vessels and high blood pressure, and it has also been suggested to account for some of the metabolic defects and neuropathological hallmarks in AD through insulin resistance, typical of type 2 diabetes [203, 204]. In this context, a meta-analysis has linked *CD2AP* to end-stage renal disease in patients with type 1 diabetes [205] (see Fig. 4).

ApoE genotype has been associated with both kidney disease and AD. People with the ApoE4 allele have an increased risk of developing AD while ApoE2 carriers are protected from the disease [32]. Conversely, in the kidney, ApoE2 is associated with reduced renal function (linked to hyperlipidemia) whereas ApoE4 attenuates the risk of end-stage renal disease, independently of race and diabetic conditions [206, 207]. *CD2AP* rs9349407 SNP modulates the association between ApoE protein plasma levels and brain amyloidosis [208]. CD2AP also regulates ApoER2 receptor levels and signaling [36] that mediates the effects of ApoE ligands. Thus, CD2AP may affect ApoE biology in both kidney and brain, with potentially divergent outcomes.

Several mechanisms have been proposed to explain how CKD leads to cognitive deficits (reviewed in [189]). The most obvious involves changes the composition of blood-borne factors (ex.: increase in uraemic compounds, decrease in neurotrophins, changes in mineral composition) reaching the brain through renal afferents and/or by altering blood–brain barrier and ultimately, affecting brain homeostasis and neuronal plasticity. Innate and adaptive immunity also play a central role in the kidney-brain axis with blood-derived immune cells impacting the function of both organs. Deregulation in the number and activity of B and T cells [209, 210] and neutrophils and monocytes [211] observed in CKD can contribute to blood–brain barrier leakage and neuronal demise via the circulatory system. Similarly, immunological defects are a core component of AD pathogenesis [212–214]. In this perspective, CD2AP loss of function in specific cell types of the kidney and brain has been shown to promote inflammation in these organs [36, 79] accompanied with fragilization of the slit diaphragm and blood–brain barrier, respectively [36, 100].

The molecular signaling framework found in podocyte and particularly in juxtaposed pedicels of the kidney that comprises proteins like dendrin, neurexin, endophilin, dynamin and synaptojanin (etc.) shows striking similarities in terms of organization and functionality with that of the neuronal synapse (Fig. 4). In podocytes, CD2AP interacts with endophilin, dynamin and synaptojanin that are critical for endosomal trafficking [71]. These proteins are also active at neuronal synapses where CD2AP is found and has been linked to altered trafficking [63]. As endocytic trafficking and protein degradation are major pathways disturbed in CKD and AD, *CD2AP* genetic variants may cause similar damages in both kidney and brain.

CD2AP is important for Aβ production in the brain (see section above) but it could also help to clear the peptide based on its newly discovered role in the brain endothelium [36]. After Aβ passes from the brain to the blood through the blood–brain barrier or other pathways, the kidney then clears circulating Aβ: Human urine contains soluble Aβ [215] and I^125^-labelled Aβ injected in the brain is excreted in the urine [216]. Furthermore, patients with CKD show abnormally elevated levels of plasma Aβ40 and Aβ42, and lower CSF levels of Aβ40 and Aβ42 [217, 218]. Conversely, in mutant APP mice, the accumulation of Aβ in the brain alters protein expression in the kidney [219]. Taken together, these findings further strength the ideas of a bidirectional kidney <—> brain effect of Aβ and that altered kidney-mediated Aβ clearance may contribute to AD neuropathology.

In support of this idea of a kidney-brain axis centered on CD2AP is the recent and exciting finding that α-synuclein accumulates in the kidney of CKD patients (17 out of 20 patients) and Lewy body diseases (10 out of 11 patients) [220]. Physiologically, in male mice, α-synuclein in the blood is quickly degraded and removed in the kidney with minimal amounts excreted in the urine. Upon renal failure, a mouse model of Parkinson’s disease injected with α-synuclein fibrils intravenously, displays worsened motor deficits and pathology [220]. Remarkably, the spreading of α-synuclein from the kidney to the brain was abrogated by denervation of renal afferents [220], suggesting the existence of nerve fibers/small vessels interface and kidney-brain axis in α-synucleopathy. Altogether, these results reveal that the kidney removes α-synuclein from the blood and kidney disease conditions can determine the extent of motor impairment and proteinopathy. Whether renal CD2AP is important for clearance of Aβ (or another toxic molecule) and synergizes with CD2AP in the brain to produce AD hallmarks remains to be determined. The existence of a kidney-brain axis for α-synucleopathy, combined with the central roles of CD2AP in kidney and brain, and the presence of Aβ in the urine, are solid reasons to investigate this avenue of research.

## Conclusions

Although CD2AP has been the subject of extensive research in kidney pathology, its role as an adaptor protein is still an emerging and less understood subject in AD. Here, we have reviewed the physiology of CD2AP and discussed how its genetic variants and gene dosage experiments in mice have informed us on potential pathogenic roles in AD. From recent studies, microglial CD2AP is emerging as an immune and neuronal regulator through microglial activation and hippocampal synapse phagocytosis, respectively. Its neuronal form also modulates tau and Aβ toxicity with implication for protein accumulation, propagation and cell signaling. For instance, endothelial CD2AP regulates the brain vessel response to Aβ in a sex-dependent manner and, more broadly, controls cerebrovascular dynamics that are critical for memory function. Altogether, the findings point to various cell-type specific functions for CD2AP and consolidate the importance of the protein in AD pathogenesis.

Despite these advances, many key questions remain unanswered. As *CD2*AP gene expression is minimal in murine microglia but prominent in human microglia, CD2AP functions must be clarified in this cell type, in a species-dependent manner. This is critical considering that microglia cells are advanced to be central in the pathogenesis of AD including in Aβ toxicity, tau accumulation, inflammation and synaptic loss. While male mice and human subjects appear to be more sensitive to loss of CD2AP function in brain vessels, determining the role of brain endothelial CD2AP in cognition of female mice constitutes an important experiment to fully better understand sex differences in AD. Building on this perspective, future multi-omics analysis of human cohorts must take into consideration the biological sex factor, the provenance of the tissue (entorhinal vs parietal cortex vs hippocampus) and percentage ApoE4 genotype carriers. For example, considering that endothelial cell gene expression profiles are heterogenous across brain regions [221], the location of the sampling could generate different results. The impact of the *CD2AP* polymorphisms still remains unclear and therefore, modeling these variants in mice or iPSCs in a cell-type specific manner would be valuable additions to the current gene dosage experiments. Moving forward, studying CD2AP and any other AD genetic factor must take into consideration the concept of brain-body interactions, especially if the protein is expressed widely in the human body and/or accumulates in peripheral organs. Investigating the roles of CD2AP in the kidney-brain axis, as done for α-synuclein [220], may shed new light onto the CD2AP mechanisms in AD. Along the same idea, as the renal function can modify the concentrations of the AD blood biomarkers such Aβ, tau and Neurofilament Light Chain [222–225], *CD2AP* genetic variants may impact the composition of these biomarkers in the urine.

Certainly, sex differences observed in mice and humans are present in the peripheral immune system, the brain vasculature and neuronal/glial networks, etc. [226–231]. In humans, around 40% of the genes display sex differences in regulation in at least one tissue [232]. Therefore, analyses based on biological sex differences at molecular, cellular, system, behavioral and organismal levels will be required in AD, as in any other complex neurodegenerative disorders [230, 233–236]. Ultimately, this combined new knowledge will allow refined therapeutic strategy designs based on kidney-brain axis and biological sex differences. Overall, the evidence reviewed highlights CD2AP as a central nexus connecting cerebral, vascular, and renal pathologies, all involved in the progression of AD. The next challenge will lie in developing targeted treatments to restore and optimize the cellular pathways involving CD2AP in these diseases.

## Data Availability

No datasets were generated or analysed during the current study.

## Abbreviations

Aβ: Amyloid-beta
AD: Alzheimer’s disease
APP: Amyloid precursor protein
ApoE4: Apolipoprotein E epsilon 4
ApoER2: Apolipoprotein E receptor 2
BACE-1: Beta-site APP cleaving enzyme 1
CD2AP: CD2-associated protein
CBF: Cerebral blood flow
CKD: Chronic kidney disease
CSFR1: Colony stimulating factor 1
eNOS: Endothelial Nitric Oxide Synthase
FSGS: Focal segmental glomerulosclerosis
GWAS: Genome-Wide Association Studies
LRP8: Lipoprotein receptor-related protein 8
ICAM-1: Intercellular Adhesion Molecule 1
NK: Natural killer
RTK: Receptor tyrosine kinase
SNPs: Single nucleotide polymorphisms (s)
SORL1: Sortilin Related Receptor 1
SH3: Src-Homology 3
